# The effectiveness and risks of Treating people with Idiopathic Pulmonary fibrosis with the Addition of Lansoprazole (TIPAL): study protocol for a randomised placebo-controlled multicentre clinical trial

**DOI:** 10.1136/bmjopen-2024-088604

**Published:** 2025-02-05

**Authors:** Megan Jones, Anthony Cahn, Nazia Chaudhuri, Allan B Clark, Ian Forrest, Matthew Hammond, Stephen Jones, Toby M Maher, Helen Parfrey, Ganesh Raghu, A John Simpson, Jaclyn Ann Smith, Lisa G Spencer, David Thickett, Luke Vale, Shajahan Wahed, Christopher Ward, Andrew M Wilson

**Affiliations:** 1 Norwich Clinical Trials Unit, University of East Anglia, Norwich, UK; 2 GlaxoSmithKline Plc, Brentford, UK; 3 Ulster University, Coleraine, UK; 4 Norwich Medical School, University of East Anglia, Norwich, UK; 5 Royal Victoria Infirmary, The Newcastle Upon Tyne Hospitals NHS Foundation Trust, Newcastle Upon Tyne, UK; 6 Action for Pulmonary Fibrosis, Peterborough, UK; 7 Keck School of Medicine of University of Southern California, Los Angeles, California, USA; 8 Papworth Hospital NHS Foundation Trust, Cambridge, Cambridgeshire, UK; 9 Center for Interstitial Lung Diseases, University of Washington Medical Center, Seattle, Washington, USA; 10 Translational and Clinical Research Institute, Newcastle University, Newcastle upon Tyne, UK; 11 Division of Infection, Immunity and Respiratory Medicine, University of Manchester, Manchester, UK; 12 Thoracic Medicine, Aintree University Hospitals NHS Foundation Trust, Liverpool, UK; 13 University of Birmingham School of Clinical and Experimental Medicine, Birmingham, UK; 14 Health Economics Group, Institute of Health and Society, Newcastle University, Newcastle, UK; 15 Newcastle Upon Tyne Hospitals NHS Trust, Newcastle Upon Tyne, UK

**Keywords:** Pulmonary Disease, Computed tomography, Clinical Trial

## Abstract

**Introduction:**

Idiopathic pulmonary fibrosis (IPF) is a chronic progressive fibrotic lung disease frequently complicated by gastro-oesophageal reflux disease. Although several observational studies and a pilot study have investigated the role of proton pump inhibitors (PPIs) in IPF, their efficacy is unknown and there is much debate in international IPF guidelines on their use. We aim to undertake an adequately powered double-blind placebo-controlled randomised multicentre clinical trial to assess the change in forced vital capacity (FVC), cough and other important patient-reported outcomes, following 12-month therapy with PPIs in people with IPF.

**Methods and analysis:**

A total of 298 patients with IPF diagnosed by a multidisciplinary team according to international guidelines who are not receiving PPIs will be enrolled. Patients are randomised equally to receive two capsules of lansoprazole or two placebo capsules, two times per day for 12 months. The primary outcome for the trial is change in FVC, measured at home, between the first week and last week of the study period. Secondary assessments include cough frequency (in a subgroup) measured using the VitaloJAK cough monitor, the King’s Brief Interstitial Lung Disease questionnaire, the Raghu Scale for Pulmonary Fibrosis, Medical Research Council dyspnoea score, EQ-5D-5L, Leicester Cough Questionnaire, modified DeMeester reflux symptoms questionnaire and opportunistically captured routine lung function measurements. High-resolution CT scoring will be undertaken in a subgroup. The trial is designed to determine whether treating people with IPF with lansoprazole will reduce the reduction in FVC over a year. The COVID-19 pandemic required the study to be undertaken as a remote trial.

**Ethics and dissemination:**

This study received ethical approval from the East of England Cambridgeshire and Hertfordshire Research Ethics Committee (reference 20/EE/0043; integrated research application system number 269050). Trial results will be published in a peer-reviewed journal upon completion.

**Trial registration number:**

ISRCTN13526307; ClinicalTrials.gov NCT04965298.

**STRENGTHS AND LIMITATIONS OF THIS STUDY**

- Increased flexibility, inclusivity and convenience for trial participants due to the decentralised trial design.
- Decreased burden and demand on physical resources for local site teams due to remote data collection.
- Evolution of new ways of working for the site and central teams, with both working together to conduct study assessments, required a new dynamic to be established but has proven both effective and vital to the trial’s success.
- Substantial increase in the volume of data being collected compared with the original design. Participants may monitor/review their own spirometry data themselves at home if they wish.
- Unexpected additional work for the trial team to revise the study design and coordinate central study assessments.

## Introduction

 Idiopathic pulmonary fibrosis (IPF) is a chronic fibrotic interstitial lung disease (ILD) of unknown cause with a poor prognosis and limited treatment options. People with this condition experience progressive breathlessness and a socially isolating cough which is particularly difficult to treat. They frequently have comorbid disease, gastro-oesophageal reflux disease (GORD) being one of the most common,^1^ with a correlation between radiological evidence of lung fibrosis and oesophageal reflux episodes.^2^ Multiple genes are upregulated in both IPF and GORD,^3^ and two separate recent bidirectional Mendelian randomisation studies concluded that GORD increases the risk of IPF but that IPF has no effect on GORD risk.^4 5^

Proton pump inhibitors (PPIs) are the first-line treatments for people with GORD.^6 7^ However, there is much debate about their role in IPF, with earlier systematic reviews reporting an overall reduction in all-cause mortality^8^ or IPF-related mortality^9^ with antireflux therapy, a finding not replicated in a more recent review.^10^ However, the underlying evidence base that these reviews can draw upon is limited. There has only been one randomised controlled trial of a PPI in people with IPF (PPIPF)^11^ which sampled 45 participants. It showed that a definitive large-scale trial was feasible but invasive assessment of GORD was not. There was a suggestion of a meaningful improvement in objective cough scores but no difference in patient-reported outcomes or lung physiology.^11^ PPIs have anti-inflammatory, antioxidant, and antifibrotic properties demonstrated in vitro^12^ and in vivo^13^ and may reduce disease progression in addition to their antiacid effects.^14^ However, PPIs have recognised adverse effects most notably an increased risk of community-acquired pneumonia,^15^ osteoporosis^16^ and *Clostridium difficile*-associated diarrhoea.^17^ Recent review articles have recommended an adequately powered clinical trial to investigate PPIs in people with IPF.^18 19^

The study described here was designed to answer the research question identified by the National Institute of Health Research (NIHR) Health Technology Assessment programme as part of its commission brief (No 18/14). The study was initially designed in May 2018, approved for funding in May 2019 and submitted for ethical review in January 2020 with revisions submitted in April 2020. The study design was similar to contemporaneous research protocols at the time including the use of change in forced vital capacity (FVC) as the primary endpoint, to be undertaken in hospital or clinical research facility lung function laboratories at 3 monthly intervals. FVC is regarded as a clinically meaningful endpoint for phase III clinical trials^20^ and the most appropriate option given that mortality is an impractical endpoint.^21^ FVC is accepted by the US Food and Drug Administration (FDA) as an appropriate endpoint for licensing of medication^22^ and is recommended in consensus statements.^23 24^ However, spirometry was considered to be an aerosol generating procedure (https://www.artp.org.uk/News/artp-guidance-respiratory-function-testing-and-sleep-services-during-endemic-covid-19), and as a result, provision for undertaking laboratory FVC measurements was stopped during the COVID-19 pandemic.

We had planned hospital-based assessments with face to face written informed consent, paper-based questionnaire completion and nurse-led setting up of the cough monitor as well as the laboratory lung function testing. However, at the beginning of 2020, nearly all non-COVID-19 face to face research studies were stopped due to the risks of spreading the virus and also to prioritise clinical work and COVID-19 research.^25^ Furthermore, people with IPF were considered to be clinically vulnerable^26 27^ and were advised to remain at home. It was evident that the study had to be redesigned to be a home-based study including the use of electronic consent, domiciliary spirometry, self-administered cough and activity monitoring, plus home delivery of the investigational medicinal product (IMP).

## Methods and analysis

### Aims

The primary aim of the study is to determine whether lansoprazole reduces disease progression in terms of change in FVC measured at home in patients with IPF compared with standard care, as defined by the National Institute for Health and Care Excellence (NICE) guidelines.^28^ Secondary aims are to assess the impact on cough frequency, health-related, ILD-related and cough-related quality of life, breathlessness, laboratory lung function, hospitalisation, unplanned hospital-free survival, sleep quality, reflux symptoms and high-resolution CT (HRCT) imaging scores. No concurrent economic evaluation was planned as part of the study due to the low cost of PPI. This will be the first adequately powered randomised trial of PPIs in people with IPF.

### Trial design

**Figure 1:**
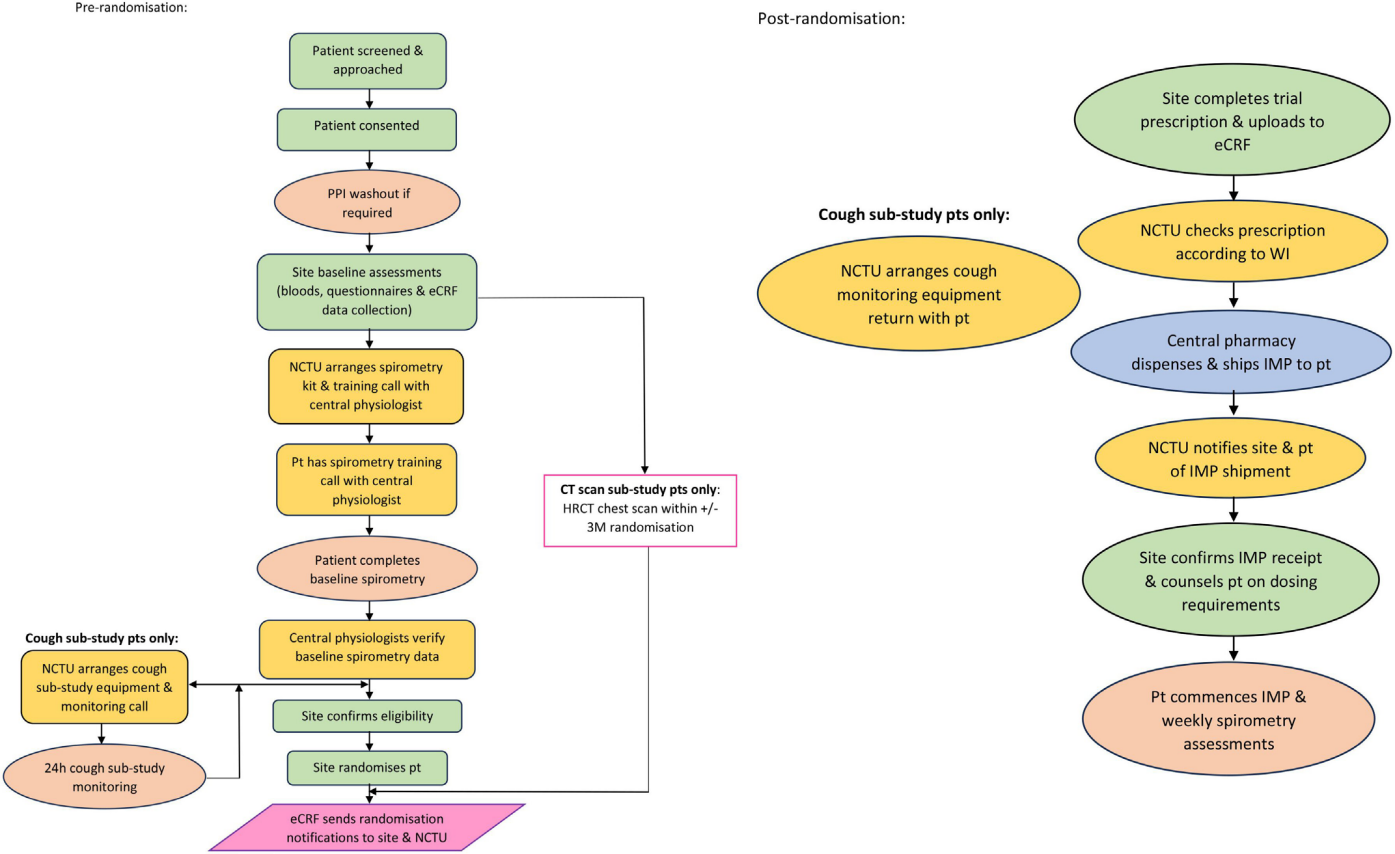
TIPAL trial design. Flowcharts presenting an overview of the prerandomisation and postrandomisation tasks for TIPAL participants. Key: green steps=site led process; yellow steps=Norwich Clinical Trials Unit-led process; orange=participant process; pink=eCRF process/randomisation; blue=central pharmacy process; grey=follow-up visits to be conducted as per protocol. eCRF: electronic case report form, CT: computed tomography, h: hour, HRCT: high resolution computed tomography, M: month, IMP: investigational medicinal product, NCTU: Norwich Clinical Trials Unit, PPI: proton pump inhibitor, Pt: patient

The study is a phase III double-blind, parallel group, 1:1 randomised, placebo-controlled, multicentre, clinical superiority trial of oral lansoprazole versus placebo in 298 participants with IPF diagnosed by multidisciplinary team (MDT) meeting consensus, according to international criteria for IPF, with outcomes being assessed during a treatment period of 12 months. There is an optional cough substudy with monitoring of cough frequency, sleep and physical activity; an optional imaging substudy with assessment of HRCT scanning; and a study within a trial (SWAT) to explore patient support group-facilitated recruitment and engagement. Figure 1 provides a study flowchart of trial design and online supplemental table 1 provides the schedule of assessments.

The Norfolk and Norwich University Hospitals NHS Foundation Trust is the trial sponsor and has delegated responsibility for the overall management of the trial to the Chief Investigator and Norwich Clinical Trials Unit (NCTU). The identification, screening and enrolment logs, linking participant-identifiable data to a pseudoanonymised participant identification number, are held locally by the research sites. Participants provide written informed consent for NCTU staff to have access to their contact details for the purposes of delivering the study and providing updates about the trial. Trial data are recorded, using the participant identification number, on an electronic case report form developed using Microsoft Visual Basic.NET/ASP.NET 2012 and Microsoft Structured Query Language server. Remote monitoring is being undertaken. If a participant withdraws from the study, the data and samples acquired prior to that point will be retained. A data management plan has been developed, which contains further information on data collection and cleaning, and will be reviewed and updated during the trial.

The PURPOSE Study is a SWAT, designed to evaluate the potential of patient support groups to improve recruitment and retention rates in clinical trials. This cluster randomised trial, registered on the Northern Ireland Network for Trials Methodology Research registry on 21 September 2020 (reference: SWAT 132), involves the identification, training and support of research champions within patient support groups. Support groups affiliated to research sites are randomised to receive early training at the beginning of the study or receive training that is delayed for 12 months. Support group research champions received a 1-hour training session each week for 4 weeks covering topics of the general context of their role, pulmonary fibrosis research, the TIPAL study design and empowerment. These were coordinated and supported by Action for Pulmonary Fibrosis. Support groups were given supporting materials and resources but were encouraged to make these bespoke to their needs. They were invited to brainstorm as a group and share ideas for the duration of the study. A mixed methods analysis assesses recruitment and retention into the study, hits on the TIPAL website, participants’ research experience and general research awareness of the support groups. Focus groups are being used to explore the support champions’ views of the initiative.

### Patient and public involvement

There are patient and public involvement representatives on both the Trial Management Group (TMG) and Trial Steering Committee and thus help guide and advise on trial conduct from a patient and public perspective. The representatives on the TMG are coapplicants and were involved with trial design from its inception and throughout.

Representatives were consulted on the development of participant facing materials including the spirometry app design. There is also a patient and public involvement representative involved in delivery of the SWAT.

Trial results will be discussed with the representatives prior to wider dissemination and/or submission of formal reports.

### Setting

The study is being conducted mainly in secondary and tertiary care hospitals within the UK. Sites are specialist ILD centres, meet the specifications required for specialist ILD centre status, or work in association with specialist centres. The study is designed to be undertaken in the community with electronic consent, shipping of IMP and study equipment to the participant’s home, domiciliary spirometry and patient-reported outcome assessments, and local safety blood assessment undertaken at the participant’s General Practitioner practice if possible. However onsite and/or paper-based patient-reported outcome assessments are an option at the participant’s request. Routine clinical outcome assessments are being captured opportunistically. The HRCT substudy is being undertaken in participating radiology departments and the SWAT is being undertaken within support groups.

### Characteristics of participants

People aged greater than or equal to 40 years are being entered into the trial. They are considered to have IPF based on local or regional multidisciplinary consensus according to the latest international guidelines.^29^ Patients may be receiving licensed antifibrotic medication assuming they were on a stable dose for at least 4 weeks prior to randomisation with no planned amendments for at least 4 weeks post randomisation. Dosing changes are permitted but starting and/or stopping antifibrotic medication is not permitted within the 4 weeks preceding and following randomisation. Participants may be rescreened if required. Patients with a pre-existing diagnosis of persistent cough (defined as troublesome for more than 8 weeks prior to study enrolment) are invited to participate in the cough substudy.

Patients cannot take part in the study if they are unable to comply with study assessments including the ability to complete reliable spirometry assessments, as spirometry assessment is the primary outcome. Participants cannot have a lower respiratory tract infection within 4 weeks of randomisation, have an allergy to the IMP or placebo contents or receive another IMP. Those receiving long-term oxygen therapy or concomitant use of a PPI, prokinetic drugs (cisapride, domperidone, metoclopramide, erythromycin, prucalopride etc.) or histamine-2 receptor antagonists (including over the counter medications) within 2 weeks prior to randomisation are excluded. However, patients receiving PPIs prior to study participation invitation may undergo a 2-week washout period immediately following consent, if clinically acceptable, with baseline assessments and subsequent randomisation into the study only if they remain asymptomatic at the end of this period. Participants with airflow obstruction (defined as forced expiratory volume in 1 s (FEV_1_)/FVC<0.7) are not eligible. Neither are those with a significant coexisting respiratory disease (defined as a respiratory condition other than IPF that exhibits a clinically relevant effect on respiratory symptoms and disease progression, as determined by the Principal Investigator (PI)). Those with a significant medical, surgical or psychiatric disease that, in the opinion of the patient’s attending physician, would affect safety or influence the study outcomes are also excluded, as are women of childbearing potential or who are lactating. Atazanavir, ketoconazole, itraconazole, tacrolimus, methotrexate and fluvoxamine are known to interact with PPIs and therefore participants receiving these treatments cannot be enrolled. An adverse effect of PPIs is hypomagnesaemia and therefore participants with hypomagnesaemia (defined as magnesium ≤0.6 mmol/L)^30^ are excluded from the study.

### Identification, recruitment and randomisation

The main method of patient identification is by review of ILD MDT meeting minutes or summaries, but is also via screening patient registries, hospital medical records and databases of research-interested patients. Potential recruits are being approached by local clinic teams and provided with a patient information sheet and given at least 24 hours to read this prior to consent. Consent is taken by appropriately trained clinicians or delegated members of staff either face to face or remotely using e-consent or paper. Following consent, patients meeting all inclusion criteria and none of the exclusion criteria (after review of their screening bloods and completion of baseline assessments) may be randomised without a subsequent visit. A PPI washout period may be required.

Randomisation is performed centrally according to a computer-generated randomisation code with the treatment group allocation sent to research pharmacists only. Minimisation is performed using Taves’ method with the factors measured at baseline comprising (1) study site, (2) baseline licensed medication for IPF (yes/no), (3) reflux symptoms (presence/absence) and (4) chronic cough status (yes/no).

## Interventions

Participants receive lansoprazole (generic) 30 mg as two capsules of 15 mg two times per day or placebo capsules as two capsules two times per day (a total of four capsules per day: two in the morning and two in the evening). Lansoprazole was overencapsulated by RenaClinical Ltd (now Eramol (UK) Ltd, Kent, UK) so that the treatments appear identical. Unblinding is available via the electronic case report form in emergency and nonemergency scenarios to enable treatment of adverse events, in the event of a suspected overdose, and/or upon participant or clinician request where appropriate. The capsules are taken 12 hours apart at least 30 min before food. This is supplied in packages providing 1 month’s supply and dispensed 6 monthly. The intervention is being shipped to the participant’s home address by the study’s central pharmacy. Participants receive a dosing card stating the required treatment schedule.

Treatment may be reduced to 15 mg (as 1× 15 mg capsule) or 1× placebo capsule two times per day (a total of two capsules per day: one in the morning and one in the evening), at least 30 min before food, in those confirmed or suspected of developing adverse reactions, including respiratory tract infection and pneumonia, *C. difficile* infection, and hypomagnesaemia defined as magnesium levels of ≤0.6 mmol/L, or at patient and/or clinician discretion. Those with moderate to severe liver impairment (defined as 7 or more points (B/C class) on the Child-Pugh score) are prescribed the reduced dose throughout their involvement in the study.

The central pharmacy is responsible for drug accountability for all sites. This includes records of IMP received at the pharmacy, IMP dispensed to participants and unused IMP. The central pharmacy is also responsible for ensuring IMP is handled and stored appropriately and dispensed accurately and for shipping IMP to each participant’s home address on a 6 monthly basis during trial participation (upon receipt of an appropriately signed prescription). Medication is couriered (or sent via another signed for delivery service) directly to the participant and a signature on receipt is required. Participants are advised to store their medication below 25°C but there may not be any temperature monitoring after IMP has been dispensed.

Compliance to study treatment is assessed in the form of returned capsule counts. All concomitant medication is recorded at baseline with any changes during participation recorded. Warfarin, digoxin and theophylline require increased monitoring of serum concentrations at the PI’s discretion.

All participants receive treatment as standard care for their IPF regardless of randomisation into this trial. Standard care is as defined by NICE clinical guideline 163^31^ including antifibrotic therapy, pulmonary rehabilitation, ambulatory oxygen therapy, transplant referral and palliative care input as appropriate. Comorbidities are identified and managed according to individual disease-specific guidelines. All participants (in the control and intervention arms) are provided with the publicly available British Digestive Disorder Charity (Guts UK) patient information leaflet about heartburn and reflux at entry into the study (or following consent for patients having a PPI washout period). This provides information about the causes, investigations and treatment for reflux including lifestyle changes. Dyspepsia is managed with lifestyle changes, reviewing the requirement for medications causing dyspepsia, and treatment with antacids and alginates in both groups as required at any time in the study. Participants still symptomatic with these treatments, or requiring PPIs for oesophagitis or duodenal ulcer, are withdrawn from the study.

## Outcomes

### Primary outcome

The primary outcome is disease progression as assessed by absolute change in % predicted FVC at 12 months post randomisation to lansoprazole or placebo. Spirometry is captured at baseline then weekly throughout the study at home. All patients are given a spirometer and computer tablet to provide the interface with the patient and the web-based platform for reviewing the results. The spirometers are European Conformity (CE) marked and were calibrated prior to shipment to ensure a 3% variability according to the European Respiratory Society (ERS)/American Thoracic Society (ATS) spirometry guidelines.^32^ Feedback is provided regarding the Grading System for FVC^32^ to encourage participants to meet Grade A criteria. Feedback is given to the patients regarding suboptimal blows including coughing. The session is terminated if there are three readings meeting Grade A criteria or if the participant undertakes eight attempts. After 5 days of readings, the quality of the spirometry is reviewed by two independent respiratory physiologists after assessing each of the volume time curves and expiratory portion of the flow volume loops. Participants must have 5 days of blows Grade C or above to be included into the study.

### Secondary outcomes

The following secondary outcomes are assessed comparing lansoprazole with placebo:

Cough frequency is being measured using a VitaloJAK cough monitor (Vitalograph Buckingham, UK) over a 24-hour period at baseline and 3 months post randomisation in a subgroup. Cough counting is intuitively meaningful and acceptable for patients.^33^ The VitaloJAK is the only cough-counting device that has been properly validated in IPF,^34^ with median sensitivity of 99.8% (range 98.1–100%) (unpublished data). It has been fully commercialised by Vitalograph Ltd. and was used in the PPIPF study as well as in large multicentre studies of up to 1500 individuals (P2X3 programme, Merck Pharmaceuticals). Cough score and cough-related quality of life are assessed by a 100 mm visual analogue scale (VAS) and the Leicester Cough Questionnaire^35^ respectively at baseline, 3, 6, 9 and 12 months.

Health-related quality of life is being assessed using the King’s Brief Interstitial Lung Disease (K-BILD) health-related quality of life questionnaire^36^ and the Raghu Scale for Pulmonary Fibrosis (R-Scale-PF).^37^ The K-BILD is a 15-question self-completed patient questionnaire which has a mean score of 55 (SD 19) units in IPF and a minimum clinically important difference of 6.3 units and has a significant association with mortality.^38^ The R-Scale-PF is a five-item numerical rating scale.^37^ The K-BILD is being assessed 3 monthly and the R-Scale-PF is collected at baseline and 12 months. Breathlessness is being captured using the Medical Research Council (MRC) dyspnoea score^39^ and EQ-5D-5L^40^ is being used to calculate quality-adjusted life years over the trial follow-up period.

The modified DeMeester score (recording dysphagia, heartburn and regurgitation) is being used to capture symptoms of reflux^41^ and the short Pittsburgh Sleep Quality Index^42 43^ is being used to capture sleep quality at baseline, 3 and 12 months. The STOP-bang questionnaire^44^ is capturing risk of obstructive sleep apnoea at 12 months post randomisation. The acceptability of the study design is being measured by a study-specific, non-validated questionnaire completed at baseline and 12 months, and the experience of research is captured by the NIHR Participant in Research Experience Survey^45^ and a participant feedback questionnaire at 12 months post randomisation.

FEV_1_, FVC and diffusing capacity of the lungs for carbon monoxide (DLCO) are being captured opportunistically from hospital laboratory assessments at baseline, 3, 6 and 12 months post randomisation where possible. The difference in change in weighted reticulovascular score (WRVS) between baseline and 12 months post randomisation on HRCT will be assessed using the Brainomix e-ILD programme.

Progression-free survival (with progression defined as time from date of randomisation to week of all-cause death, lung transplant or a 10% absolute reduction in FVC % predicted from baseline and measured by domiciliary spirometry). Hospital-free survival is defined as death (from all causes) or first non-elective (all-cause) hospital admission. Respiratory-related hospital-free survival will also be assessed.

### Safety outcomes

Adverse events with particular relevance to confirmed or suspected diagnoses of respiratory tract infection and pneumonia, *C. difficile* infection, and hypomagnesaemia will be recorded at each study visit following randomisation.

### Data monitoring

An independent data monitoring committee has been established and meets 6 monthly as per the study-specific Terms of Reference available from the corresponding author. The study is also subject to audit undertaken by the sponsor.

### Sample size

A sample size of 270 individuals, 135 per group, provides 90% power to detect a minimal important difference of 4% reduction in %FVC versus placebo assuming a SD of 9%,^46^ a loss to follow-up rate of 20%^46 47^ and a significance level of 5%. However, we will randomise 298 patients to account for 10% of patients being asymptomatic.

A sample size of 160 patients provides 90% power to detect a ratio of geometric means of 0.6 for cough frequency, which is smaller than the published minimal important difference,^48^ assuming a coefficient of variation of 1 (from the PPIPF trial^11^) and a loss to follow-up rate of 30%.

For the HRCT scan substudy, a sample size of 82 participants provides 80% power to detect a 3.45% difference in WRVS at a 5% significance level, assuming a SD of 5.6 and a correlation coefficient of 0.6. We will aim to recruit up to 100 participants to allow for a 20% loss to follow-up rate.

### Statistical analysis

All analyses will be conducted according to a detailed statistical analysis plan. Analyses will be adjusted for site and the use of baseline licensed medication for IPF. The analysis populations are defined as intention to treat (all randomised individuals regardless of adherence), per protocol (if compliance is less than 85%, then a compliance-adjusted causal effect analysis will also be carried out defining compliance as taking at least 80% of study medication based on pill counts) and safety population (all patients randomised who received at least one dose of the trial treatment). In addition, if there is sufficient reduction in dose among participants, a dose-response relationship will be estimated using instrumental variable regression.

The primary outcome, absolute change in %FVC at 12 months post randomisation, will be analysed using a general linear model adjusting for the minimisation factors used in the allocation algorithm. The largest FVC value with the reproducibility according to ERS/ATS spirometry guideline grading criteria of A to C^32^ of each FVC value obtained each day over 5 days will be averaged given the day to day variability of FVC.^41^ We ask for at least three blows and up to eight blows per day. An analysis will also be undertaken adjusting for the baseline %FVC. Additional adjusted analysis may be undertaken for factors associated with the outcome. In addition, a linear mixed model will be used to combine all the postrandomisation %FVC results into a single model which will adjust for the same factors and include a patient identifier as a random effect. An interaction between group and time will also be included to assess if the effect of the intervention is constant over time or varies as time progresses.

### Secondary outcomes

#### The rate of decline in %FVC during the 12 months

This will be based on a longitudinal model with a factor for the intervention or control to represent the average change over the course of the trial. It will include a time trend to represent the decline in %FVC during 12 months in the control group and a time trend × intervention interaction to represent the additional decline in %FVC during the 12 months. If there is evidence of a nonlinear time trend, average %FVC each month will be calculated and time will be treated as categorical to ease interpretation. Different temporal correlation structures will be investigated.

#### Cough frequency

This will be based on a log-transformed cough count at 3 months. The model used will be a general linear model adjusting for the minimisation factors used in the allocation algorithm. An adjusted analysis will also be undertaken adjusting for baseline cough count. The effect size will be estimated as the geometric mean.

#### Cough score, cough-related QoL, K-BILD, R-scale-PF, EQ-5D-5L, DLCO, short Pittsburgh Sleep Quality, WRVS

Analysis of these will be based on a general linear model with the value at 12 months as the outcome, adjusting for the minimisation factors used in the allocation algorithm. An analysis will also be undertaken adjusting for baseline values. The effect size will be estimated as the mean difference. In addition, a linear mixed model will be used to combine all the postrandomisation cough score results into a single model which will adjust for the same factors and include a patient identifier as a random effect. An interaction between group and time will also be included to assess if the effect of the intervention is constant over time or varies as time progresses.

#### MRC dyspnoea scale and reflux characteristics

Analysis of these will be based on a Mann-Whitney U test comparing the values at 12 months between groups. It will not be possible to adjust for the minimisation factors used in the allocation algorithm or to report an effect size; however, the median in each group will be reported. As the same analysis will be conducted at 3, 6 and 9 months, a Bonferroni adjustment will be made to the p values.

#### Sleep apnoea

The STOP-Bang questionnaire will be analysed by a low, intermediate or high risk using an ordinal logistic regression model adjusting for minimisation factors used in the allocation algorithm.

#### Progression-free survival

This will be assessed using the weekly home-based spirometry measures and hospital data. The effect size will be estimated as the HR. Cox proportional hazards will be used adjusting for the minimisation factors used in the allocation algorithm. Disease progression will be assessed from randomisation until the week of all-cause mortality, lung transplant or a 10% absolute reduction in %FVC from baseline measured by domiciliary spirometry.

#### Unplanned hospital-free survival and respiratory-related hospitalisation

These will be assessed at 3, 6, 9 and 12 months and will be presented as a number and percentage. The effect size will be estimated as the OR. Logistic regression will be used adjusting for the minimisation factors used in the allocation algorithm.

#### Study-specific questionnaire

The analysis will be descriptive, summarising the change in responses to each question from baseline.

The assumptions of all the models will be checked using residual analysis and, if appropriate, alternative methods will be used.

## Discussion

The appropriate role of antireflux therapy and PPIs in IPF is unknown. The most recent international guidelines (2022) suggest not treating patients with IPF with antacid medication for the purpose of improving respiratory outcomes,^29^ whereas the previous guidelines (which were in place when the study started) (2015) continued to recommend regular antacid treatment, such as with PPIs, for patients with IPF,^49^ as was first recommended in 2011.^50^ However, both guidelines state their recommendations are conditional and based on very low-quality evidence. The change of opinion was perhaps premature^51^ given the lack of evidence and the guideline committee awaits the result of this study to help inform the next version.^52^ The UK NICE guidelines for IPF^31^ recommend treatment for GORD as a comorbidity causing cough but do not mention PPIs as disease-modifying pharmacological intervention.

We were required to convert our primary outcome from laboratory-based lung function assessment of FVC to home spirometry assessment. Home spirometry is becoming more commonly used in clinical practice since the COVID-19 pandemic. In a 4-week study of home monitoring in the Netherlands, which consisted of daily home spirometry and online patient-reported outcomes in 12 patients with IPF, spirometry was felt to be easy and not burdensome by participants with nearly 100% adherence.^53^ Participants felt like they were in control. In one of the first studies to investigate home spirometry in people with IPF, 50 subjects performed an FVC manoeuvre daily for an average of 279 days.^54^ This study showed good acceptance of the procedure and change in FVC to be a good predictor of mortality with different patterns of decline.^54^ Weekly spirometry (three blows per procedure) was shown to have adherence of greater than 90% at least up to 24 weeks, and although there was weekly variability in at least a proportion of patients, by using weekly home spirometry measurements, it was possible to have a more efficient trial design.^55^ Despite home spirometry having been repeatedly shown to have good correlation with laboratory spirometry^53^,^55^ with correlation coefficients greater than 0.9, estimates of the rate of FVC decline obtained using home spirometry have been shown to be poorly correlated with those based on clinical spirometry.^56^ Daily spirometry has been used as an endpoint in a clinical trial of unclassifiable fibrotic interstitial lung disease, but linear regression modelling was not possible.^57^ However, in that study, only one blow per day was required, and although only those manoeuvres ‘accepted’ by the spirometry-based algorithm were considered in the analysis, ERS/ATS grading^32^ of the procedure was not possible. In our study, we ask for daily spirometry for the first week of the study and the last week of the study with weekly spirometry measurements in the intervening period. In this respect, increasing the frequency of measurements to greater than once per week does not improve the correlation.^56^ Following online video training, using the study tablet if required, by a qualified respiratory physiologist, we ask for at least three blows and up to eight blows and grade the reproducibility according to ERS/ATS criteria^32^ after review of the data by two independent respiratory physiologists with rejection of unacceptable blows.

Obtaining informed consent is fundamental to clinical research. In 2018, the UK Health Research Authority and UK Medicines and Healthcare products Regulatory Agency produced a joint statement on seeking consent by electronic methods.^58^ They advised that the participants are informed by interview in a real-time two-way communication and that consent must be ‘in writing’ which can be a typewritten signature for type A trials (those that involve risks no higher than standard medical care).^58^ However, we are collecting eSignatures that involve tracing of the participant’s hand-written signature. Participants verbally consent to the sharing of their email address to receive the link to the electronic consent form to facilitate the process. After the patient has had adequate time to understand and digest the previously mailed study information material, and following a phone/video call consultation so the researcher can ensure the patient is adequately informed, both parties complete the electronic consent form in real time on the designated field via Research Electronic Data Capture, Vanderbilt, USA. However, in order to be flexible and inclusive for those unable or unwilling to provide online eSignatures, the option of signing a hard copy of the consent form and mailing it to the researcher is acceptable after an online or telephone consultation by the researcher.^59^ The form is then countersigned, and a copy returned. Electronic consenting for conducting research remotely is generally well received by participants^60^ although the effect on enrolment into studies is unknown.^61^

The assessment of cough can be undertaken in several ways including cough frequency (captured by a 24-hour cough recording device), cough intensity (assessed by VAS) and disruption to lifestyle (measured by cough HRQoL). We are using cough monitoring as our main cough outcome given the findings from the pilot study.^11^ Cough monitoring is superior to VAS in detecting change in cough^62^ and recognised by the FDA as a key outcome in large phase III clinical trials. Cough counting is appropriate for clinical trials, and as it correlates weakly with cough intensity or cough HRQoL measures,^63^ it cannot be replaced by them. We are using the VitaloJAK cough monitor as it has been validated in IPF;^34^ however, unlike previous studies, the participants self-administer the setting up of the device at home with central support and guidance by video call.

The TIPAL study will determine whether PPIs are effective in terms of change in FVC in people with IPF who do not require these treatments for reflux disease. It will also provide information on numerous secondary endpoints, most importantly cough frequency, cough intensity and HRQoL. Given the uncertainty in international IPF guidelines, the findings will have a considerable implication for the care of people with IPF.

### Protocol amendments

We modified the protocol in August 2020 to ensure the project was deliverable during the COVID-19 pandemic. This included remote assessment of spirometry, cough frequency and questionnaires. We modified the protocol in August 2021 to permit the addition of the R-Scale-PF and in May 2022 for the capture of routine cross-sectional imaging at baseline and 12 months. In November 2022, we modified the protocol to permit a substudy to undertake HRCT images and undertake a WRVS analysis. Amendments were notified to relevant parties in line with UK trial regulations and processes.

### Trial status

The current version of the protocol is V.2.4 23 March 2023. The trial began in June 2021 and we expect recruitment to be completed by December 2025.

## Ethics and dissemination

### Ethics approval and consent to participate

The East of England Cambridgeshire and Hertfordshire Research Ethics Committee (reference 20/EE/0043) approved the trial at all participating centres (integrated research application system number 269050). Participant consent is obtained prior to any trial-related procedure. During the consent process, it is made clear that the participant can decline to participate in all or any aspect of the trial, at any time and for any reason, without affecting their future care or treatment. Patients unable to provide written informed consent are deemed ineligible for the trial. The Informed Consent Form is provided as online supplemental material.

Trial results will be published in a peer-reviewed journal upon study completion.

## supplementary material

10.1136/bmjopen-2024-088604 online supplemental file 1
