# Supplementary material for: The effectiveness and risks of Treating people with Idiopathic Pulmonary fibrosis with the Addition of Lansoprazole (TIPAL): study protocol for a randomised placebo-controlled multicentre clinical trial
Source: BMJ Open. 2025 Feb 5;15(2):e088604. doi: 10.1136/bmjopen-2024-088604 (PMC11800218; doi:10.1136/bmjopen-2024-088604)
Supplement: online supplemental file 1 [file bmjopen-15-2-s001.docx]

**Table 1**. Schedule of Assessments.

|  | **Screening^1^** | | **Baseline^1^** | **Randomisation** | **3 Months^2, 3^**  **(+/- 4 weeks)** | **6 Months^3, 4^**  **(+/- 4 weeks)** | **9 Months^3, 4^**  **(+/-4 weeks)** | **12 Months^3,4^**  **(+/-4 weeks)** |
| --- | --- | --- | --- | --- | --- | --- | --- | --- |
| **Informed Consent** | X | |  |  |  |  |  |  |
| **Eligibility** | X | |  |  |  |  |  |  |
| **Demographics, medical history, and patient characteristics** |  | | X |  |  |  |  |  |
| **Standard care CT scan collected/new scan performed^4^** |  | | X |  |  |  |  | X |
| **Randomisation** |  | |  | X |  |  |  |  |
| **IMP dispensed** |  | |  | X |  | X |  |  |
| **IMP adherence** |  | |  |  |  | X |  | X |
| **Weekly domiciliary spirometry** | Undertaken **weekly throughout** trial | | | | | | | |
|  | **Screening^1^** | **Baseline^1^** | | **Randomisation** | **3 Months^2, 3^**  **(+/- 4 weeks)** | **6 Months^3, 4^**  **(+/- 4 weeks)** | **9 Months ^3,4^**  **(+/-4 weeks)** | **12 Months^3, 4^**  **(+/-4 weeks)** |
| **Cough count sub-study** |  | X | |  | X |  |  |  |
| ***Leicester Cough Questionnaire, MRC Dyspnoea Scale, K-BILD, EQ-5D-5L & cough score questionnaires*** |  | X | |  | X | X | X | X |
| ***Study-specific questionnaire and R-Scale-PF*** |  | X | |  |  |  |  | X |
| ***STOP-Bang Questionnaire, NIHR PRES & feedback questionnaire^5^*** |  |  | |  |  |  |  | X |
| ***Short Pittsburgh Sleep Quality Index and modified DeMeester Score*** |  | X | |  | X |  |  | X |
| **Adverse events** |  |  | |  | X | X | X | X |
| **Safety bloods (full blood count, urea and electrolytes, liver function tests, calcium, and magnesium)^6^** |  | X | |  | X^10^ | X^10^ | X^10^ | X |
| **Blood sample for genotype analysis^7^** |  | X | |  | X | X | X | X |
| **Research bloods^8^** |  | X | |  |  |  |  | X |
| **Lung function (including spirometry & gas transfer) assessments where possible^9^** |  | X | |  | X | X |  | X |

^1^ Where participants are not attending in person consent and collection of up-to-date trial data not available in the patient’s notes take place remotely via phone/video call. Questionnaires are completed and returned by freepost/courier/electronically. Safety bloods are taken at GP surgeries, according to local policy for remote bloods, or site.

^2^ Where participants are not attending in person, adverse events are recorded during phone/video call with questionnaires completed and returned by freepost/courier/electronically and safety bloods taken at GP surgeries, according to local policy for remote bloods, or site. Cough count sub-study monitoring are conducted remotely via phone/video call or in person at 3 months for cough sub-study participants only.

^3^ Visits should take place within 4 weeks either side of scheduled dates.

^4^Standard care chest HRCT scans are collected from all trial participants. Patients consenting to the CT scan sub-study undergo additional chest HRCT scans at baseline and/or 12 months, if no standard care scans are available, provided they are willing. **CT scan sub-study scans are performed within 3 months (+/-) of randomisation and within 3 months (+/-) of the 12 months timepoint.**

^5^ TIPAL participants are asked to complete the NIHR PRES and participant feedback questionnaire at 12 months.

^6^ 10mL blood must be taken for safety analyses at baseline and 12 months. **Baseline bloods are acceptable within 6 months of randomisation assuming no change to the patient’s clinical condition, at the discretion of the PI**.

^7^ 10mL blood are taken for genotype analysis **once at any timepoint.**

^8^ 20mL blood are taken for research blood analyses at baseline and 12 months where possible.

^9^ This refers to laboratory-based lung function (including spirometry and gas transfer) assessments conducted as part of **standard care** only.

^10^ Safety bloods at 3, 6 and 9 months are taken only if deemed necessary by the PI or sub-I, due to a relevant change in the participants’ clinical status.

NB where study assessments are completed within 28 days of randomisation for baseline or within the timeframes specified above as part of standard care, these observations can be recorded at the relevant time point to avoid patients having to repeat assessments unnecessarily, provided they adhere to the requirements of the study protocol.

Baseline questionnaires are acceptable within 6 weeks of randomisation.

Participants are permitted to repeat baseline FVC measurements upon receipt of the domiciliary spirometer to familiarise themselves with use of the equipment. Baseline FVC measurements should be attempted after a successful PPI washout (where required), for a period up to a maximum of 28 days upon which a decision as to whether to proceed to trial enrolment is made by the local PI in collaboration with the chief investigator and patient where appropriate if clinically consistent results have been challenging to obtain. Baseline and 12-month domiciliary spirometry measurements are repeated daily for 5 days.
