# Supplementary material for: The effectiveness and risks of Treating people with Idiopathic Pulmonary fibrosis with the Addition of Lansoprazole (TIPAL): study protocol for a randomised placebo-controlled multicentre clinical trial
Source: BMJ Open. 2025 Feb 5;15(2):e088604. doi: 10.1136/bmjopen-2024-088604 (PMC11800218; doi:10.1136/bmjopen-2024-088604)
Supplement: Supplementary file 2 [file bmjopen-2024-088604.reviewer_comments.pdf]

PEER REVIEW HISTORY

BMJ Open publishes all reviews undertaken for accepted manuscripts. Reviewers are asked to complete a checklist review form and are provided with free text boxes to elaborate on their assessment. These free text comments are reproduced below.

ARTICLE DETAILS

Title (Provisional)

The effectiveness and risks of Treating people with Idiopathic Pulmonary fibrosis with the Addition of Lansoprazole (TIPAL): study protocol for a randomised placebo-controlled multi-centre clinical trial

Authors

Jones, Megan; Cahn, Anthony; Chaudhuri, Nazia; Clark, Allan B; Forrest, Ian; Hammond, Matthew; Jones, Stephen; Maher, Toby M; Parfrey, Helen; Raghu, Ganesh; Simpson, A John; Smith, Jaclyn Ann; spencer, lisa G; Thickett , David; Vale, Luke; Wahed, Shajahan; Ward, Christopher; Wilson, Andrew M

VERSION 1 - REVIEW

**Reviewer**	**1**
**Name**	**Ruaro, Barbara**
**Affiliation**	**University of Trieste**
**Date**	**25-May-2024**
**COI**	**None**

The paper is quite well written.The article covers a very interesting and current topic. Nevertheless, in my opinion, some parts need to be improved, I have some comments:

1) Abstract 5 Introduction

6 Although there are a large number of observational studies and a small pilot study investigating the 7 role of proton pump inhibitors (PPIs) in idiopathic pulmonary fibrosis (IPF), the efficacy of these drugs 8 is unknown and there is much debate in International IPF Guidelines. We aim to undertake an 9 adequately powered double-blind placebo-controlled randomised multicentre clinical trial of PPIs to 10 assess the change in forced vital capacity (FVC), cough, and other important patient reported 11 outcomes. Please, improve this paragraph and add some information on IPF and GERD

2) Introduction 2 Idiopathic pulmonary fibrosis (IPF) is a chronic fibrotic interstitial lung disease (ILD) of unknown cause 3 with a poor prognosis and limited treatment options. People with this condition experience 4 progressive breathlessness and a socially isolating

cough which is particularly difficult to treat. They 5 frequently have comorbid disease, gastro-oesophageal reflex disease (GORD) being one of the most 6 common(1), with a correlation between radiological evidence of lung fibrosis and oesophageal reflux 7 episodes(2). Two separate recent bidirectional Mendelian randomisation studies concluded that 8 GORD increases the risk of IPF but that IPF has no effect on GORD risk(3, 4). I suggest to improve this part by discussing some latest references than these (1. Raghu G, Freudenberger TD, Yang S, Curtis JR, Spada C, Hayes J, et al. High prevalence of 3 abnormal acid gastro-oesophageal reflux in idiopathic pulmonary fibrosis. Eur Respir J. 4 2006;27(1):136-42.

5 2. Savarino E, Carbone R, Marabotto E, Furnari M, Sconfienza L, Ghio M, et al. Gastro6 oesophageal reflux and gastric aspiration in idiopathic pulmonary fibrosis patients. Eur Respir J.

7 2013;42(5):1322-31) such as:

- Gastroesophageal Reflux Disease in Idiopathic Pulmonary Fibrosis: Viewer or Actor? To Treat or Not to Treat?. Pharmaceuticals (Basel). 2022;15(8):1033. Published 2022 Aug 22. doi:10.3390/ph15081033

- The Role of Proton Pump Inhibitors in the Realm of Idiopathic Pulmonary Fibrosis and its Associated Comorbidities: A Systematic Review. Cureus. 2024;16(3):e55980. Published 2024 Mar 11. doi:10.7759/cureus.55980

- Prediction and analysis of genetic effect in idiopathic pulmonary fibrosis and gastroesophageal reflux disease. IET Syst Biol. 2023;17(6):352-365. doi:10.1049/syb2.12081

3) Methods and Analysis

22 Aims

23 The primary aim of the study is to determine whether lansoprazole reduces disease progression in

24 terms of change in FVC measured at home in patients with IPF compared to standard care, as defined

25 by the National Institute for Health and Care Excellence (NICE) guidelines(24). Secondary aims are to

26 compare the clinical efficacy in terms of cough count, health-, ILD- and cough-related quality of life .... Please, improve the descriptio of study aim to clarify the novelty of this research.

4) Sample Size

2 A sample size of 270 individuals, 135 per group, provides 90% power to detect a minimal important 3 difference of 4% reduction in % FVC versus placebo assuming a standard

deviation of 9%(39), a loss to 4 follow-up rate of 20%(39, 40), and a significance level of 5%. However, we will randomise 298 patients

5 to account for 10% of patients being asymptomatic. Please, the inclusion and the exclusion criteria.

5) Discussion

9 The appropriate role of anti-reflux therapy and PPIs in IPF is unknown. The most recent international 10 guidelines suggest not treating patients with IPF with antacid medication for the purpose of improving 11 respiratory outcomes(25) whereas the previous guidelines (which were in place when the study 12 started) recommended regular antacid treatment for patients with IPF(43). However, both guidelines 13 state their recommendations are conditional and based on very low-quality evidence. The change of 14 opinion was perhaps premature(44) given the lack of evidence and the guideline committee awaits

15 the result of this study to help inform the next version (45). The discussion section needs to be improved. It could be interesting to improve the decsription of the aim of the study. It is necessary to clarify the novelty of the study and compare it with published literature.

**Reviewer**	**2**
**Name**	**Morgan, Ann**
**Affiliation**	**National Heart and Lung Institute, Respiratory Epidemiology**
**Date**	**07-Jun-2024**
**COI**	**none**

General comments

I read this trial protocol with great interest and found it to be comprehensive and very readable. I have just a few minor comments and suggestions for clarifications.

Specific comments

Methods

Primary outcome: Given the importance of the weekly domiciliary spirometry to the trial, I would have expected to see some comments around the validity/reliability of the devices that are being used in this trial, perhaps in the form of manufacturers'/published evidence relating to the performance of the chosen devices. Would such information be available? I note that the broader issue of the reliability and acceptability of devices for home spirometry is covered in the Discussion.

Discussion

Para 1: Just for reader clarity, it might be helpful to include the actual year of the various international guidelines relating to the management of IPF (2015 and 2022). It might also be worth be more explicit about how the recommendations given in the international guidelines with respect to the use of PPIs in IPF have changed and possibly to what extent this advice has been embodied in UK/NICE guidance and clinical practice.

[From a 2021 BMJ review: Although the 2015 ATS/ ERS/JRS/ALAT IPF treatment guidelines give a conditional recommendation for the use of antacid therapy, even in patients without symptoms of gastroesophageal reflux, the 2022 updated guideline makes a conditional recommendation against its use for the purpose of improving respiratory outcomes.]

Para 2: While I cannot profess to be an expert in this area, I did wonder whether the picture painted in this paragraph of the reliability of home spirometry in terms of monitoring FVC change was perhaps a little one sided. There are likely other studies which are not quite as positive (one example below), and perhaps there needs to be some attempt to be a little more balanced. However, I do note that you do mention steps taken to ensure that measurements are as accurate as possible.

From Noth I, et al (data from the INMARK trial). Eur Respir J. 2021 Jul 8;58(1):2001518
[Home spirometry was a feasible and valid measure of lung function in patients with IPF and preserved FVC, but estimates of the rate of FVC decline obtained using home spirometry were poorly correlated with those based on clinic spirometry.]

VERSION 1 - AUTHOR RESPONSE

Response to Reviewer 1:

1) Abstract Introduction
Although there are a large number of observational studies and a small pilot study investigating the role of proton pump inhibitors (PPIs) in idiopathic pulmonary fibrosis (IPF), the efficacy of these drugs is unknown and there is much debate in International IPF Guidelines. We aim to undertake an adequately powered double-blind placebo-controlled randomised multicentre clinical trial of PPIs to assess the change in forced vital capacity (FVC), cough, and other important patient reported outcomes. Please, improve this paragraph and add some information on IPF and GERD

We have improved the wording of this paragraph and added some information about IPF and GERD.

2) Introduction Idiopathic pulmonary fibrosis (IPF) is a chronic fibrotic interstitial lung disease (ILD) of unknown cause with a poor prognosis and limited treatment options. People with this condition experience progressive breathlessness and a socially isolating cough which is particularly difficult to treat. They frequently have comorbid disease, gastro-oesophageal reflex disease (GORD) being one of the most common(1), with a correlation between radiological evidence of lung fibrosis and oesophageal reflux episodes(2). Two separate recent bidirectional Mendelian randomisation studies concluded that 8 GORD increases the

risk of IPF but that IPF has no effect on GORD risk(3, 4). I suggest to improve this part by discussing some latest references than these (1. Raghu G, Freudenberger TD, Yang S, Curtis JR, Spada C, Hayes J, et al. High prevalence of 3 abnormal acid gastro-oesophageal reflux in idiopathic pulmonary fibrosis. Eur Respir J. 4 2006;27(1):136-42.
5 2. Savarino E, Carbone R, Marabotto E, Furnari M, Sconfienza L, Ghio M, et al. Gastro6 oesophageal reflux and gastric aspiration in idiopathic pulmonary fibrosis patients. Eur Respir J.
7 2013;42(5):1322-31) such as:
- Gastroesophageal Reflux Disease in Idiopathic Pulmonary Fibrosis: Viewer or Actor? To Treat or Not to Treat?. Pharmaceuticals (Basel). 2022;15(8):1033. Published 2022 Aug 22. doi:10.3390/ph15081033
- The Role of Proton Pump Inhibitors in the Realm of Idiopathic Pulmonary Fibrosis and its Associated Comorbidities: A Systematic Review. Cureus. 2024;16(3):e55980. Published 2024 Mar 11. doi:10.7759/cureus.55980
- Prediction and analysis of genetic effect in idiopathic pulmonary fibrosis and gastroesophageal reflux disease. IET Syst Biol. 2023;17(6):352-365. doi:10.1049/syb2.12081

Thank you for highlighting these additional references. It would not be appropriate to replace the current citations as they are review articles, however, they do highlight the need for this clinical trial and we have references them as such. We have included the reference regarding genetic association.

3) Methods and Analysis
Aims
The primary aim of the study is to determine whether lansoprazole reduces disease progression in terms of change in FVC measured at home in patients with IPF compared to standard care, as defined by the National Institute for Health and Care Excellence (NICE) guidelines(24). Secondary aims are to compare the clinical efficacy in terms of cough count, health-, ILD- and cough-related quality of life .... Please, improve the descriptio of study aim to clarify the novelty of this research.

We have modified the wording revised for clarity and added a sentence regarding novelty of research added as suggested.

4) Sample Size
A sample size of 270 individuals, 135 per group, provides 90% power to detect a minimal important difference of 4% reduction in % FVC versus placebo assuming a standard deviation of 9%(39), a loss to follow-up rate of 20%(39, 40), and a significance level of 5%. However, we will randomise 298 patients to account for 10% of patients being asymptomatic. Please, the inclusion and the exclusion criteria.

The eligibility criteria are outlined in the 'Characteristics of Participants' section.

5) The appropriate role of anti-reflux therapy and PPIs in IPF is unknown. The most recent international guidelines suggest not treating patients with IPF with antacid medication for the purpose of improving respiratory outcomes(25) whereas the previous guidelines (which were in place when the study 12 started) recommended regular antacid treatment for patients with IPF(43). However, both guidelines state their recommendations are conditional

and based on very low-quality evidence. The change of opinion was perhaps premature(44) given the lack of evidence and the guideline committee awaits the result of this study to help inform the next version (45). The discussion section needs to be improved. It could be interesting to improve the description of the aim of the study. It is necessary to clarify the novelty of the study and compare it with published literature.

We have highlighted the importance of the study in the aims as requested and made a comparison with the published literature.

Response to Reviewer 2:

1 Primary outcome: Given the importance of the weekly domiciliary spirometry to the trial, I would have expected to see some comments around the validity/reliability of the devices that are being used in this trial, perhaps in the form of manufacturers'/published evidence relating to the performance of the chosen devices. Would such information be available? I note that the broader issue of the reliability and acceptability of devices for home spirometry is covered in the Discussion.

We have stated that the spirometer used is CE marked and given greater detail about the method for ensuring the quality of the spirometry data.

2 Discussion. Para 1: Just for reader clarity, it might be helpful to include the actual year of the various international guidelines relating to the management of IPF (2015 and 2022). It might also be worth be more explicit about how the recommendations given in the international guidelines with respect to the use of PPIs in IPF have changed and possibly to what extent this advice has been embodied in UK/NICE guidance and clinical practice.

We have added the year of guidelines and also a statement about the current clinical practice as per the UK national guidelines.

3 Discussion. Para 2: While I cannot profess to be an expert in this area, I did wonder whether the picture painted in this paragraph of the reliability of home spirometry in terms of monitoring FVC change was perhaps a little one sided. There are likely other studies which are not quite as positive (one example below), and perhaps there needs to be some attempt to be a little more balanced. However, I do note that you do mention steps taken to ensure that measurements are as accurate as possible.

We agree with the reviewer that there are data showing the challenges of home spirometry. We had presented the issues with home spirometry in a previous clinical and also made reference to the paper by Noth et al. However we have modified the manuscript to highlight the lack of relationship between home and laboratory spirometry identified by Noth et al.

VERSION 2 - REVIEW

Reviewer 1

Name **Ruaro, Barbara**

Affiliation **University of Trieste**

Date **20-Nov-2024**

COI

The manuscript has been improved, as requested. No further comments.

Reviewer **2**

Name **Morgan, Ann**

Affiliation **National Heart and Lung Institute, Respiratory Epidemiology**

Date **09-Dec-2024**

COI

Second round review comments

Reviewers' previous comments seem to be adequately addressed by the authors and I have no further substantive comments.

The text may however benefit from a final critical copy edit to ensure that there are no outstanding areas of repetition or potential ambiguity. The abstract for instance could be more transparent about how the trial design evolved over time (due to outbreak of the COVID pandemic) and the chronology. Recruitment is under way and expected to be complete by the end of 2024? It might also be helpful to spell out the eligibility criteria in terms of GORD status? Patients must not be "receiving PPIs" but can have symptoms of acid reflux; does this mean people with a diagnosis of GORD are excluded? What about people who self-medicate, i.e. may take over-the-counter PPIs on a more ah hoc basis?

VERSION 2 - AUTHOR RESPONSE

Reviewer's comments 1 (19/06/2024)

Reviewer: 1

Comments to the Author:

The paper is quite well written. The article covers a very interesting and current topic. Nevertheless, in my opinion, some parts need to be improved, I have some comments:

1) Abstract - Introduction

Although there are a large number of observational studies and a small pilot study investigating the role of proton pump inhibitors (PPIs) in idiopathic pulmonary fibrosis (IPF), the efficacy of these drugs is unknown and there is much debate in International IPF Guidelines. We aim to undertake an adequately powered double-blind placebo-controlled randomised multicentre clinical trial of PPIs to assess the change in forced vital capacity (FVC), cough, and other important patient reported outcomes. Please, improve this paragraph and add some information on IPF and GERD

2) Introduction

Idiopathic pulmonary fibrosis (IPF) is a chronic fibrotic interstitial lung disease (ILD) of unknown cause with a poor prognosis and limited treatment options. People with this condition experience progressive breathlessness and a socially isolating cough which is particularly difficult to treat. They frequently have comorbid disease, gastro-oesophageal reflex disease (GORD) being one of the most common(1), with a correlation between radiological evidence of lung fibrosis and oesophageal reflux episodes(2). Two separate recent bidirectional Mendelian randomisation studies concluded that GORD increases the risk of IPF but that IPF has no effect on GORD risk(3, 4). I suggest to improve this part by discussing some latest references than these (Raghu G, Freudenberger TD, Yang S, Curtis JR, Spada C, Hayes J, et al. High prevalence of 3 abnormal acid gastro-oesophageal reflux in idiopathic pulmonary fibrosis. Eur Respir J. 4 2006;27(1):136-42. Savarino E, Carbone R, Marabotto E, Furnari M, Sconfienza L, Ghio M, et al. Gastro6 oesophageal reflux and gastric aspiration in idiopathic pulmonary fibrosis patients. Eur Respir J. 2013;42(5):1322-31) such as:

- Gastroesophageal Reflux Disease in Idiopathic Pulmonary Fibrosis: Viewer or Actor? To Treat or Not to Treat?. Pharmaceuticals (Basel). 2022;15(8):1033. Published 2022 Aug 22. doi:10.3390/ph15081033

- The Role of Proton Pump Inhibitors in the Realm of Idiopathic Pulmonary Fibrosis and its Associated Comorbidities: A Systematic Review. Cureus. 2024;16(3):e55980. Published 2024 Mar 11. doi:10.7759/cureus.55980

- Prediction and analysis of genetic effect in idiopathic pulmonary fibrosis and gastroesophageal reflux disease. IET Syst Biol. 2023;17(6):352-365. doi:10.1049/syb2.12081

3) Methods and Analysis

Aims

The primary aim of the study is to determine whether lansoprazole reduces disease progression in terms of change in FVC measured at home in patients with IPF compared to standard care, as defined by the National Institute for Health and Care Excellence (NICE) guidelines(24). Secondary aims are to compare the clinical efficacy in terms of cough count, health-, ILD- and cough-related quality of life .... Please, improve the descriptio of study aim to clarify the novelty of this research.

4) Sample Size

A sample size of 270 individuals, 135 per group, provides 90% power to detect a minimal important difference of 4% reduction in % FVC versus placebo assuming a standard deviation of 9%(39), a loss to follow-up rate of 20%(39, 40), and a significance level of 5%. However, we will randomise 298 patients to account for 10% of patients being asymptomatic. Please, the inclusion and the exclusion criteria.

5) Discussion

The appropriate role of anti-reflux therapy and PPIs in IPF is unknown. The most recent international guidelines suggest not treating patients with IPF with antacid medication for the purpose of improving

respiratory outcomes(25) whereas the previous guidelines (which were in place when the study started) recommended regular antacid treatment for patients with IPF(43). However, both guidelines state their recommendations are conditional and based on very low-quality evidence. The change of opinion was perhaps premature(44) given the lack of evidence and the guideline committee awaits the result of this study to help inform the next version (45). The discussion section needs to be improved. It could be interesting to improve the decsription of the aim of the study. It is necessary to clarify the novelty of the study and compare it with published literature

Response: Thank you for your comments. The manuscript was updated as follows to address these comments:

1) Abstract introduction revised with text added for context and to provide further details on the study.

2) The introduction was revised following review of more recent publications as suggested. Bibliography updated as required.

3) The description of the secondary outcomes was updated for clarity and text added to highlight the novelty of this research.

4) Eligibility criteria outlined in the 'characteristics of participants' section of the manuscript within original submission.

5) Discussion updated in line with the suggestions made by both reviewers at this stage. The manuscript now reflects the changing landscape regarding proton pump inhibitor prescription and outlines the novelty of this trial.

<u>Reviewer: 2</u>

Comments to the Author:

General comments

I read this trial protocol with great interest and found it to be comprehensive and very readable. I have just a few minor comments and suggestions for clarifications.

Specific comments

Methods

Primary outcome: Given the importance of the weekly domiciliary spirometry to the trial, I would have expected to see some comments around the validity/reliability of the devices that are being used in this trial, perhaps in the form of manufacturers'/published evidence relating to the performance of the chosen devices. Would such information be available? I note that the broader issue of the reliability and acceptability of devices for home spirometry is covered in the Discussion.

Discussion

Para 1: Just for reader clarity, it might be helpful to include the actual year of the various international guidelines relating to the management of IPF (2015 and 2022). It might also be worth be more explicit about how the recommendations given in the international guidelines with respect to the use of PPIs in IPF have changed and possibly to what extent this advice has been embodied in UK/NICE guidance and clinical practice.

[From a 2021 BMJ review: Although the 2015 ATS/ ERS/JRS/ALAT IPF treatment guidelines give a conditional recommendation for the use of antacid therapy, even in patients without symptoms of gastroesophageal reflux, the 2022 updated guideline makes a conditional recommendation against its use for the purpose of improving respiratory outcomes.]

Para 2: While I cannot profess to be an expert in this area, I did wonder whether the picture painted in this paragraph of the reliability of home spirometry in terms of monitoring FVC change was perhaps a little one sided. There are likely other studies which are not quite as positive (one example below), and perhaps there needs to be some attempt to be a little more balanced. However, I do note that you do mention steps taken to ensure that measurements are as accurate as possible.

From Noth I, et al (data from the INMARK trial). Eur Respir J. 2021 Jul 8;58(1):2001518 [Home spirometry was a feasible and valid measure of lung function in patients with IPF and preserved FVC, but estimates of the rate of FVC decline obtained using home spirometry were poorly correlated with those based on clinic spirometry.]

Response: Thank you for your comments. The manuscript was updated as follows to address these comments.

- Primary outcome section updated to provide further details on domiciliary spirometry data collection and study-specific processes being followed.
- Discussion revised in line with both reviewers comments including broadening the references reviewed to give a more balanced view of the current literature and additional context to the changing clinical guidelines to date. Bibliography updated as required.

Reviewer's comments 2 (10/12/2024)

Reviewer: 1

Comments to the Author:

The manuscript has been improved, as requested. No further comments.

Response: Thank you. No further amendments made as a result.

Reviewer: 2

Comments to the Author:

Second round review comments

Reviewers' previous comments seem to be adequately addressed by the authors and I have no further substantive comments.

The text may however benefit from a final critical copy edit to ensure that there are no outstanding areas of repetition or potential ambiguity. The abstract for instance could be more transparent about how the trial design evolved over time (due to outbreak of the COVID pandemic) and the chronology. Recruitment is under way and expected to be complete by the end of 2024? It might also be helpful to spell out the eligibility criteria in terms of GORD status? Patients must not be "receiving PPIs" but can have symptoms of acid reflux; does this mean people with a diagnosis of GORD are excluded? What about people who self-medicate, i.e. may take over-the-counter PPIs on a more ah hoc basis?

Response: Thank you for your suggestions. Minor amendments to correct typographical errors, remove the repetition and/or ambiguity, include a reference to over-the-counter proton pump inhibitors, and an update to the expected completion date have been made. Patient GORD status is

not explicitly referenced in the trial eligibility criteria, and as such reference to it as not been made here. We have reviewed the abstract and do not think amendments are required as the trial chronology is explained with the body of the manuscript. These changes are shown in the tracked changes copy v3.2 17/12/2024 uploaded onto ScholarOne. No further changes made.
